# Broad Spectrum Activity of a Lectin-Like Bacterial Serine Protease Family on Human Leukocytes

**DOI:** 10.1371/journal.pone.0107920

**Published:** 2014-09-24

**Authors:** Jorge Luis Ayala-Lujan, Vidhya Vijayakumar, Mei Gong, Rachel Smith, Araceli E. Santiago, Fernando Ruiz-Perez

**Affiliations:** 1 Department of Pediatrics, University of Virginia School of Medicine, Charlottesville, Virginia, United States of America; 2 Unidad Academica de Ciencias Quimicas, Universidad Autonoma de Zacatecas, Zacatecas, Mexico; 3 Department of Immunology and Microbiology, University of Maryland at Baltimore, Baltimore, Maryland, United States of America; INRA Clermont-Ferrand Research Center, France

## Abstract

The serine protease autotransporter from Enterobacteriaceae (SPATE) family, which number more than 25 proteases with apparent diverse functions, have been phylogenetically divided into two distinct classes, designated 1 and 2. We recently demonstrated that Pic and Tsh, two members of the class-2 SPATE family produced by intestinal and extraintestinal pathogenic *E. coli*, were able to cleave a number of O-glycosylated proteins on neutrophils and lymphocytes resulting in impaired leukocyte functions. Here we show that most members of the class-2 SPATE family have lectin-like properties and exhibit differential protease activity reliant on glycoprotein type and cell lineage. Protease activity was seen in virtually all tested O-glycosylated proteins including CD34, CD55, CD164, TIM1, TIM3, TIM4 and C1-INH. We also show that although SPATE proteins bound and cleaved glycoproteins more efficiently on granulocytes and monocytes, they also targeted glycoproteins on B, T and natural killer lymphocytes. Finally, we found that the characteristic domain-2 of class-2 SPATEs is not required for glycoprotease activity, but single amino acid mutations in Pic domain-1 to those residues naturally occurring in domain-1 of SepA, were sufficient to hamper Pic glycoprotease activity. This study shows that most class-2 SPATEs have redundant activities and suggest that they may function as immunomodulators at several levels of the immune system.

## Introduction

The most abundant and functionally diverse proteolytic enzymes in living organisms are the serine proteases [1]. The serine protease autotransporters from Enterobacteriaceae (SPATE) constitute a superfamily of virulence factors whose members resemble those belonging to the trypsin-like superfamily of serine proteases [2], [3]. SPATE proteins are produced by enteric pathogens including *E. coli*, *Shigella* and recently, also found in *Salmonella, Edwardsiella*, and *Citrobacter* species [2], [3]. Interestingly, SPATEs are produced by all recognized pathogenic *E. coli* strains including enteropathogenic *E. coli* (EPEC), shiga toxin-producing *E. coli* (STEC), enterotoxigenic *E. coli* (ETEC), enterohemorrhagic *E. coli* (EHEC), enteroinvasive *E. coli* (EIEC), enteroaggregative *E. coli* (EAEC), diffusely adherent *E. coli* (DAEC), adherent-invasive *E. coli* (AIEC), uropathogenic *E. coli* (UPEC) and by the animal pathogens: avian pathogenic *E. coli* (APEC) and rabbit pathogenic *E. coli* (REPEC), all agents of enteric/diarrheal disease [2], [3].

SPATEs are secreted members of the autotransporter family, whose secretion involves the excision of the N-terminal region, known as the “passenger domain”, from the C-terminal region or “β-domain domain”, and subsequent release of the passenger domain (which harbors the proteolytic activity) into the cell surroundings [2], [4].

The SPATE family, which now includes more than 25 proteases, has been phylogenetically divided into class-1 and class-2 based on the amino acid sequence of the passenger domain [2], [3], [5]. The bifurcation of SPATEs into two classes is consistent with structural differences and biological effects. Class-1 SPATEs such as Pet, SigA, Sat, and EspC for example, display similar substrate specificity, consistent cytotoxic effects on cultured cells, and enterotoxin activity on intestinal tissues (reviewed in reference [2]). On the other hand, most knowledge on class 2 SPATEs comes from two members of this family: Tsh/Hbp and the Pic protease. Tsh (Temperature-sensitive hemagglutinin), the first class-2 SPATE isolated from a septicemic pathogen in poultry [6], was found to confer hemagglutination and binding to extracellular matrix proteins, such as fibronectin and collagen IV. Subsequently, Hbp (Haemoglobin binding protein) identified in the *E. coli* strain (EB1) [7], isolated from a human wound infection, was shown to differ from Tsh in only two residues. Hbp was able to interact with hemoglobin, to degrade it, and subsequently to bind the released heme suggesting a role for Hbp in heme acquisition [7]. Pic (Protease involved in intestinal colonization) originally identified in *Shigella flexneri* 2a and EAEC [8], was found to cleave mucin from numerous sources, to induce mucus release [9], and to confer a subtle competitive advantage in mucosal colonization [10]. We recently showed that Pic and Tsh/Hb cleave a variety of leukocyte surface glycoproteins with diverse roles in numerous cellular and immune functions, and which were substituted with carbohydrates structurally similar to those found on human mucin glycoproteins [11]. More importantly, cleavage of those glycoproteins triggered adverse effects on leukocyte functions such as chemotaxis, transmigration, activation and apoptosis [11]. Therefore, we have proposed a main role for Pic and Tsh/Hbp in immune evasion [11]. Here, we show that the class-2 SPATE family shares a lectin-like property and have proteolytic activity against a large variety of O-linked glycoproteins in the hematopoietic cell lineage, and in both, innate and adaptive immunity.

**Table 1:** Primer sequences used in this study.

Primer Name	Sequence	Use
S124T sense	5′-gtatcgcctcagtatatcgtcaccgtaaagcataacg-3′	Site mutagenesis
S124T antisense	5′-cgttatgctttacggtgacgatatactgaggcgatac-3′	Site mutagenesis
N128V sense	5′-cagtatatcgtcagcgtaaagcatgtcggaggatatcgga-3	Site mutagenesis
N128V antisense	5′-tccgatatcctccgacatgctttacgctgacgatatactg-3′	Site mutagenesis
Y131S sense	5′-cagcgtaaagcataacggaggaagtcggagtgtgagctt-3	Site mutagenesis
Y131S antisense	5′-aagctcacactccgacttcctccgttatgctttacgctg-3′	Site mutagenesis
N138Y sense	5′-ggaatatgtatttttcccataaccaaagctcacactccga-3′	Site mutagenesis
N138Y antisense	5′-tcggagtgtgagctttggttatgggaaaaatacatattcc-3′	Site mutagenesis
L145A sense	5′-aagggtggttattacggtcaacagcggaatatgtatttttcccattac-3′	Site mutagenesis
L145A antisense	5′-gtaatgggaaaaatacatattccgctgttgaccgtaataaccaccctt-3′	Site mutagenesis
S153G sense	5′-tggagcatggaagtcaataccagggtggttattacggtca-3′	Site mutagenesis
S153G antisense	5′-tgaccgtaataaccaccctggtattgacttccatgctcca-3′	Site mutagenesis
G253L sense	5′-aacggccctttacctgactatttagcccctgggg-3′	Site mutagenesis
G253L antisense	5′-ccccaggggctaaatagtcaggtaaagggccgtt-3′	Site mutagenesis
pBADadcA sense	5′-ccggaccggtaaggcgagctccccgttttttgggctaacaggaggaattaaccatgaataa aatatatgcgataaaaaagaac-3′	AdcA cloning
pBADadcA antisense	5′-ttaaggtaaggtctcctcgagtcagaatgaataacggatattagcatta-3′	AdcA cloning
pBADcrc2sp sense	5′-ccggaccggtaaggcgagctccccgttttttgggctaacaggaggaattaaccatgaata aaatatactcgctgaaatactgt-3′	Crc2sp cloning
pBADcrc2 antisense	5′-ttaaggtaaggtctcctcgagtcagaacatataacggaagttcgcg-3′	Crc2sp cloning

## Materials and Methods

Recombinant proteins and antibodies. Human glycosylated recombinant proteins: C1-INH, CX3CL, CD44, CD45, CD55, CD97, CD99, CD162, TIM1, TIM3, TIM4, fibronectin, E-selectin and human Integrin alpha M beta 2 were obtained from R&D systems Inc. (Minneapolis, MN 55413), while the human glycosylated recombinant proteins: CD34, CD43, CD93, and CD164 were obtained from Sino Biologicals (Beijing 100176 P. R. China). Antibodies used in this study were dye conjugated mouse anti human CD3, CD4, CD8, CD14, CD16, CD56, CD43, CD44, CD45 and CD162 from BD and Invitrogen). Rabbit anti-sialic acid antibody was purchased from Cloud Clone Corp, Houston, TX.

### Cell lines and human primary cells

Binding experiments and immunofluorescence assays were performed in the Jurkat T cell line (Clone E6-1, American Type Culture Collection, Manassas, VA,20110. USA). Jurkat cells were routinely cultured in RPMI 1640 medium, supplemented with 10% heat inactivated fetal bovine serum (FBS) and antibiotics: 100 U/ml penicillin, and 100 µg/ml streptomycin at 37°C in a humidified 95% air and 5% CO2. Fresh human blood to isolate primary cells were purchased from Allcells LLC. (Alameda, CA 94502).

### Leukoagglutination assays

Jurkat cells were incubated in 48-well plates at a concentration of 5×10^5^ cells/200 µL in RPMI medium containing 10% FBS and incubated at 37°C and CO_2_ with different SPATEs and observed in an inverted microscope at 1 and 3 hrs.

### Deglycosylation of glycoproteins

Glycoproteins were deglycosylated with deglycosylation enzyme cocktail (New England BioLab) under denaturing and non-denaturing conditions, and following the manufacturer’s recommendations. Denaturing conditions: briefly, 20 µg of glycoproteins in denaturing buffer were denatured by heating at 100°C for 10 minutes. Denatured proteins were treated with G7 reaction buffer, NP40, and 5 µl deglycosylation enzyme cocktail [Neuraminidase (12,500), *O*-Glycosidase (10000000 units), β1-4-Galactosidase (2000 units), β-N-acetylglucosaminidase (1000 units), PNGase-F (125000 units)], and incubated overnight at 37°C. Non denaturing conditions: 20 µg of glycoproteins in G7 reaction buffer were treated with 5 ul of deglycosylation enzyme cocktail, mixed gently and incubated overnight at 37°C. To determine the extent of deglycosylation by both protocols, samples were analyzed by mobility shifts on SDS-PAGE gels.

### Immunofluorescence assays

Following incubation of Jurkat cells with SPATEs, cells were washed and suspended in flow cytometry buffer (HBSS without Ca2++/Mg2++ and 0.2% BSA) and blocked with human IgG. Then, cells were incubated for 1 hour with FITC anti-CD43 and PE anti-CD162, washed twice with FACS buffer and fixed with 2.5% formalin. 10 µL were extended on slides and allowed to dry out, and counterstained with ProLong Gold antifade reagent with DAPI (Invitrogen). Samples were analyzed in a Leica fluorescence microscope.

### Binding of SPATEs to leukocytes

To determine binding of SPATEs to different leukocyte subpopulations, SPATEs were labeled with FITC (Pierce Biotechnology). Therefore, 1 mg/mL of each SPATE were incubated with 100 µg/mL FITC in 0.1 M sodium carbonate buffer (pH 9.6) for 1 hour at room temperature. Using desalting columns from the same manufacturer, FITC-labeled SPATE (SPATE-FITC) were separated from unbound FITC. For binding of SPATEs-FITC to leukocytes, 1 million total leukocytes were incubated with increasing concentrations of SPATE-FITC in HBSS/1% BSA buffer for 30 minutes on ice. Alternatively, cells were incubated with heat-denatured SPATE labeled with FITC or a combination of equimolar concentrations of unlabeled SPATE and SPATE-FITC. SPATE binding on specific cell types was identified through “forward and side scatter” gates plus cell type-specific lineage markers including anti-CD3, -CD14, -CD16, -CD19, and -CD56( [12]) (Figure S1). After washing the cells twice with HBSS/1% BSA, fluorescence was measured on a flow cytometer (Beckmann Coulter Cyan ADP LX. Brea, California) and data were analyzed with FloJo version 4.5 (TreeStar, Ashland, OR).

### Preparation of total leukocyte cells

Fresh heparinized blood from healthy human donors purchased from AllCells LLC. (Alameda, CA 94502) was mixed 1∶1 with dextran/0.9% NaCl (final concentration, 1.5% dextran) and allowed to settle undisturbed for 30 min. To remove residual erythrocytes, the cell fraction from the leukocyte-rich plasma was spun down at 1500 rpm/15 min and resuspended in 12 ml of cold sterile water for 25 seconds followed by addition of 4 ml of 3% NaCl. Red cell debris was removed by centrifugation at 1500 rpm/10 min, followed by a wash with HBSS buffer without Ca++/Mg++ (Invitro gene). Total leukocytes (this fraction included lymphocytes, NK, monocytes and granulocytes) were enumerated by using a hemocytometer.

### Purification of SPATE proteins

Expression and purification of SPATE proteins from minimal clones in *E. coli* HB101 have been described previously [13]. Anion-exchange purified proteins were treated with Endo-Trap-Blue (Hyglos GmbH. 82347 Bernried am Starnberger See Germany) to remove LPS accordingly to the manufacturer’s specifications, dialyzed in 1× PBS pH 7.0, and stored in small aliquots at −80°C.

### Construction of pBAD30-Crc2sp and pBAD30-AdcA

Most of the SPATE minimal clones used in this study were published previously [13], except the Crc2sp and AdcA clones, which were constructed in this study. The crc2sp (NCBI accession No. YP_003368482.1) [2] and adcA (NCBI accession No. YP_003367548.1 [2], [14], genes were amplified by PCR from the *C. rodentium* genome (American Type Culture Collection, Manassas, VA,20110. USA) using Pfx platinum DNA polymerase (Invitrogen) and primers in Table-1. The amplification products were digested with SacI and XhoI restriction enzymes and cloned under the pBAD promoter in the pBAD30 plasmid previously digested with SacI and SalI enzymes. The PCR cycling conditions were as follows: denaturing at 95°C for 5 min; followed by 30 cycles of denaturing at 94°C for 2 min, annealing at 60°C for 30 s, and extension at 68°C for 2 min; and a final cycle of 68°C for 10 min.

### Site-directed mutagenesis

Site-directed mutagenesis was performed following the QuikChange protocol (Stratagene, Cedar Creek, TX) and with the PfuTurbo (Stratagene) high-fidelity polymerase. The pACYC184-Pic template was used at 25 to 50 ng per reaction with 10 pmol of each of the complementary primers. Reactions were carried out according to the manufacturer’s protocol. Primers used to generate the single point mutations are shown in table-1. All constructs were verified by sequencing at the University of Virginia DNA Core Facility.

## Results

### Phylogenetic analysis of the Class-2 SPATE family

**Figure 1:**
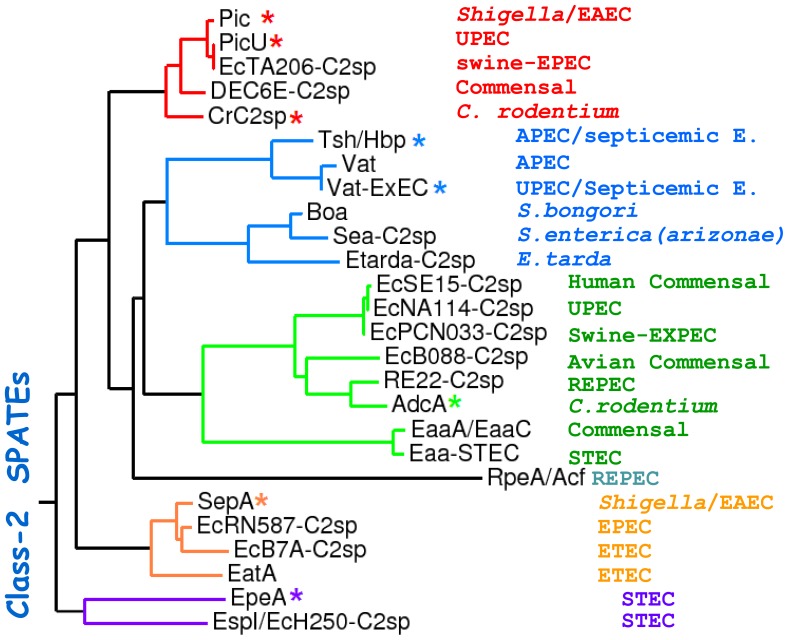
Sequence relationships of class-2 SPATE proteins. The phylogenetic analysis of the aminoacidic sequence of class-2 SPATEs shown here was modified from reference [2], with permission of the Editorial board. The phylogenetic tree shows allelic variants of SPATEs in clusters (colored branches) Bacterial species and pathotypes from which SPATE sequences were derived are shown on the right side. Class-2 SPATEs also include a cluster of proteases found mostly in animal pathogens, which lack of the classic domain-2 (green branches, see text). SPATE members of each major cluster purified in this study are indicated with asterisks.

The SPATE family has been divided into two classes. Figure-1 shows an updated phylogenetic tree of the class-2 SPATE subfamily (reviewed in [2]). Members of this class show high degree of allelic variation and are grouped into clusters (Figure 1, colored branches). Among class-2 SPATEs, there is a subset of hypothetical allelic SPATE variants that do not exhibit the characteristic N-terminal protein protuberance termed domain-2 [15], exhibited by Tsh/Hbp and many other class-2 SPATEs, and which are mainly distributed among animal pathogens (Figure 1, green branch). In order to determine whether the class-2 family share similar properties with Pic and Tsh/Hbp, we purified one or more members of each major phylogenetic cluster (Figure 1, indicated with an asterisk) including Pic, PicU, Crc2sp, Tsh/Hbp, Vat-Ex, AdcA, EpeA and SepA from minimal clones in *E. coli* HB101. PicU and Crc2sp share 95.7/97.6% and 74.6/85.0% identity/similarity to Pic, respectively; AdcA and Vat-Ex share 45.6/62.4% and 69.6/79.2% identity/similarity to Tsh/Hbp, respectively. SepA and EpeA still belonging to the class-2 SPATE family occupy more distant branches, sharing 49.5/67.4% and 43.7/62.4% identity/similarity to Pic, respectively.

### Class-2 SPATEs agglutinate white cells

**Figure 2:**
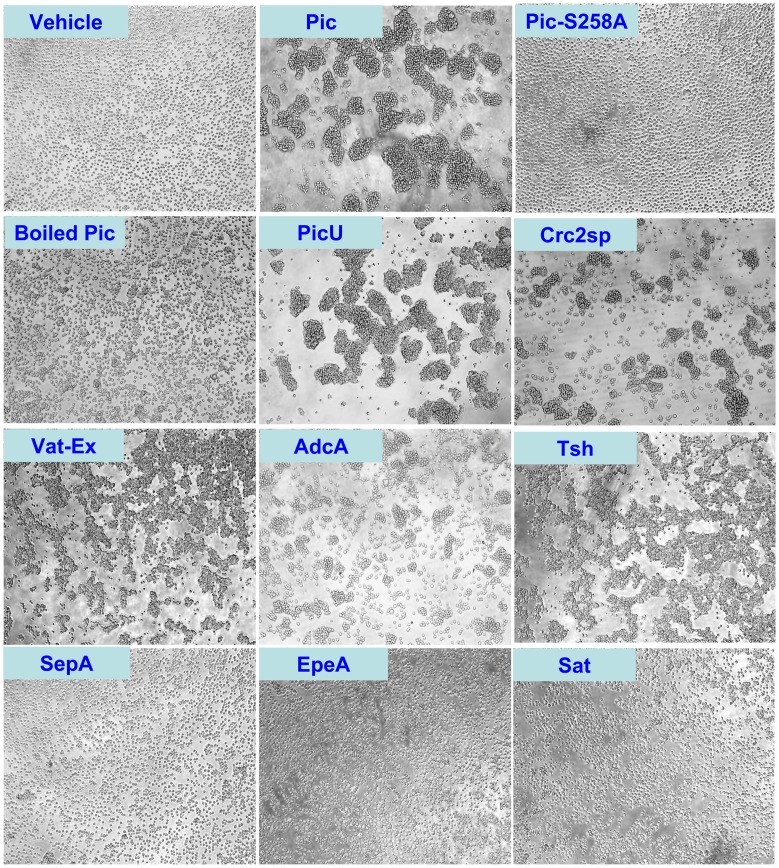
Class-2 SPATEs trigger leukoagglutination. 1×10^5^ Jurkat T cell were incubated with 2 µM of each class-2 SPATE at 37°C in a CO_2_ incubator. One hour following initial treatment, cells were analyzed in an inverted light microscopy. Micrographs were taken in the light field at 20× magnification.

Pic and Tsh have been previously shown to display lectin-like activity, agglutinating red cells and cleaving leukocyte glycoproteins. To assess agglutination ability to leukocytes, we incubated Jurkat T cells with 2 µM of purified SPATE proteins for 1 h and recorded the agglutination phenotype microscopically (Figure 2). We noticed that although most class-2 SPATEs were able to agglutinate white cells, a heat-denatured Pic, the Pic258A mutant (a protease deficient Pic), SepA and EpeA did not display this activity. Likewise, the class-1 SPATE, Sat did not show this phenotype (Figure 2). Since the agglutinating activity seemed to be a consequence of protease activity, we next looked for direct binding of SPATEs to leukocytes.

### Binding of SPATEs to human Leukocyte subpopulations

**Figure 3:**
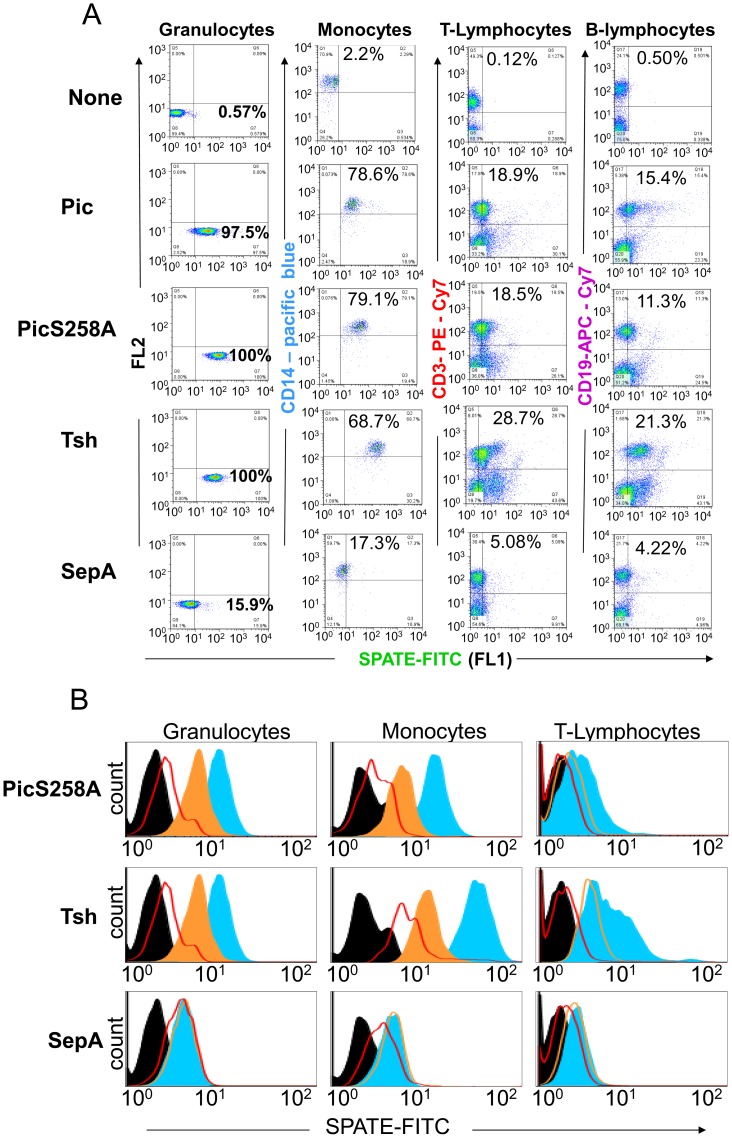
Binding of class-2 SPATEs to leukocyte subpopulations. **A**, Multicolor flow cytometry was used to analyze direct binding of class-2 SPATE members (Pic, the protease deficient PicS258A, Tsh/Hbp and SepA) to different leukocyte subpopulations. Leukocytes were incubated with 2 µM of each FITC-labeled SPATE for 30 minutes on ice. To differentiate for specific leukocyte subpopulations, monocytes, T lymphocytes, and B lymphocytes were simultaneously stained with dye-conjugated antibodies directed against CD14, CD3 and CD19, respectively. Granulocytes were only selected by gating. **B**, Inhibition of SPATE binding by heat treatment and by competitive assays. Binding of FITC-labeled PicS258A, Tsh and SepA were tested as above (shaded in blue) and in the following conditions: as FITC-denatured SPATEs (outlined in red), or in a competitive binding with equimolar concentrations (2 µM) of unlabeled versus FITC-labeled SPATEs (shaded in orange). Untreated leukocyte populations are shaded in black. Histograms of FITC-SPATE binding on leukocytes are shown. Flow cytometry data are representative of at least three independent experiments.

To further document direct binding of SPATEs to leukocytes, we incubated total human leukocytes with FITC-labeled Pic, the protease deficient Pic258A and Tsh for 30 minutes in ice. Specific cell types were identified through scatter gates and cell type-specific lineage markers by flow cytometry (Figure 3) [12]. SPATEs bound efficiently to monocytes (>68% CD14^+^) and granulocytes (>97%), whereas binding of SPATEs to T (>18%, CD3^+^) and B (>11%, CD19^+^) lymphocytes was to a lesser degree (Figure 3A). In order to determine the specificity of this binding we incubated leukocyte populations with FITC-labeled heat-denatured PicS258A, Tsh or SepA. In a separate experiment we incubated leukocytes with an equimolar combination of FITC-labeled and unlabeled SPATEs, and determined the percentage of leukocyte demonstrating FITC-SPATE binding. We observed substantial reduction of PicS258A and Tsh/Hbp binding to granulocytes and lymphocytes when incubating with denatured SPATEs or when competing with unlabeled SPATEs, while SepA showed only background binding levels to leukocytes, which was not affected when using denatured SepA or when competing with unlabeled SepA (Figure 3B).

### Broad spectrum activity of class-2 SPATEs on mucin-type O-glycoproteins of the immune system

**Figure 4:**
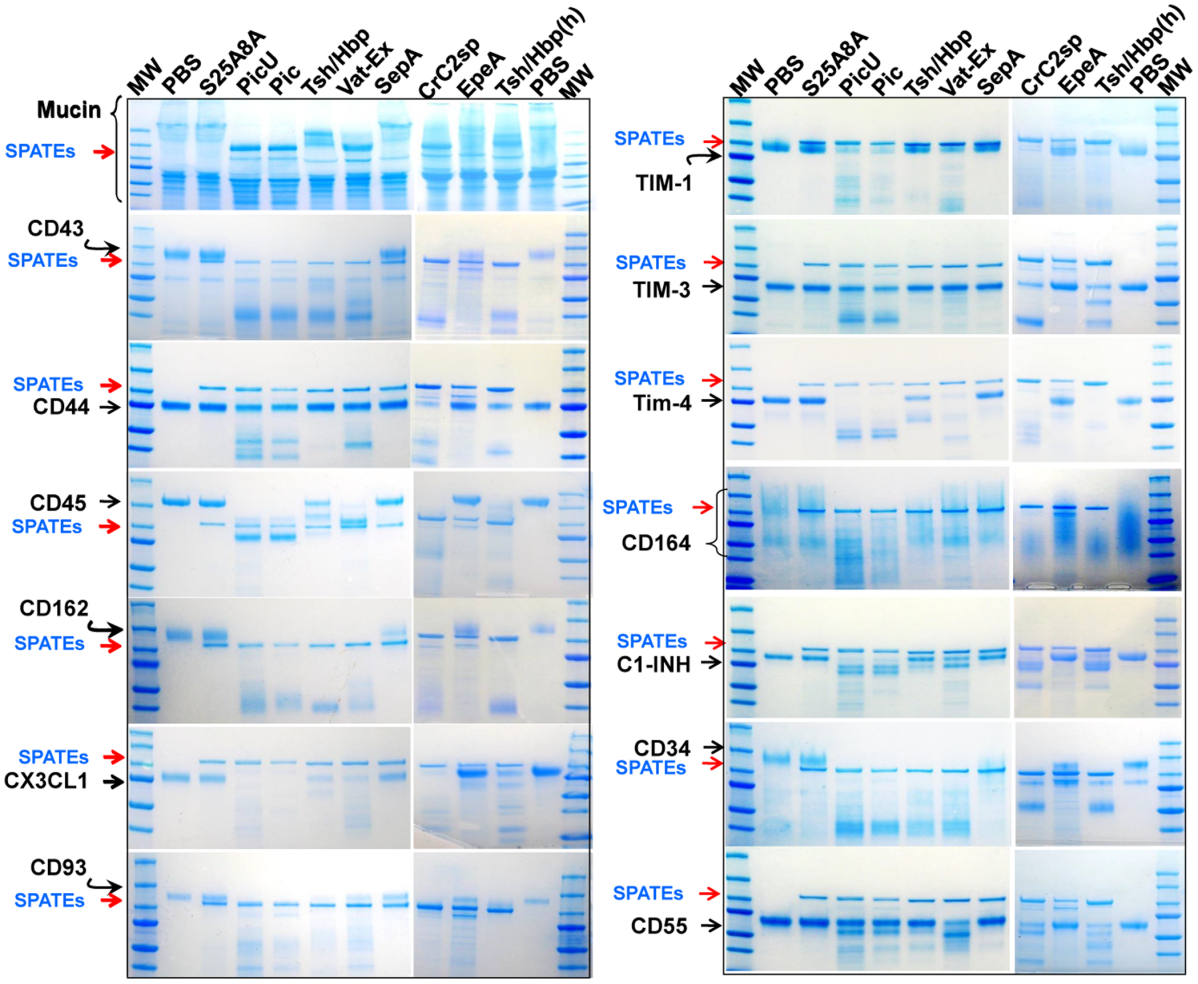
Broad spectrum activity of class-2 SPATEs on mucin-type O-glycoproteins of the immune system. 3 µg of recombinant glycosylated proteins from human origin were incubated with 2 µM of each purified SPATE at 37°C for 1 h. Samples were analyzed by 4–20% gradient SDS/PAGE and blue Commassie staining. SPATEs and intact glycoproteins are indicated with arrows. Tsh/Hbp was also tested at 4 µM indicated with (h).

We previously showed that Pic and Tsh/Hbp were able to cleave O-linked glycoproteins such as CD43, CD44, CD45, CD93, CD162 and CX3CL on human neutrophils and lymphocytes. Here we tested all other class-2 SPATEs on the same and new glycoproteins involved in diverse functions of the immune system (Figure 4). Three µg of glycoproteins were incubated with 2 µM of each SPATE for 1 h at 37°C, and analyzed by SDS-PAGE and Commassie staining. We found that most class-2 SPATEs tested except SepA, EpeA or the Pic258A mutant efficiently cleaved the substrates mentioned above, and many others including CD34, CD55, CD164, TIM1, TIM2, TIM3 and C1-INH, all of them known to possess O-linked carbohydrates (Figure 4) [16], [17], [18], [19], [20]. Pic, PicU and Crc2sp showed the most astringent protease activity as we noticed extensive cleavage in almost all glycoproteins tested. Tsh/Hbp and Vat-Ex showed comparable activities. The differential cleavage pattern seen on glycoproteins could be explained by the glycosylation level of the molecules, as some glycoproteins such as CD43, CD93, CD162 and CX3CL are known to be extensively O-glycosylated [21], [22], [23], [24], and which were almost completely digested by SPATEs, suggesting the presence of multiple cleavage sites. CD44 and CD45 are known to be just partially glycosylated reliant on isoforms [21], [25], whereas CD55 and C1-INH have limited sites of O-glycosylation [19], [20], [25]. Extensive degradation of slightly glycosylated substrates by SPATEs was not seen despite prolonged incubation times (overnight) or with higher SPATE concentrations (>2 uM). SepA, EpeA or the PicS258A did not show any protease activity under these conditions (Data not shown).

**Figure 5:**
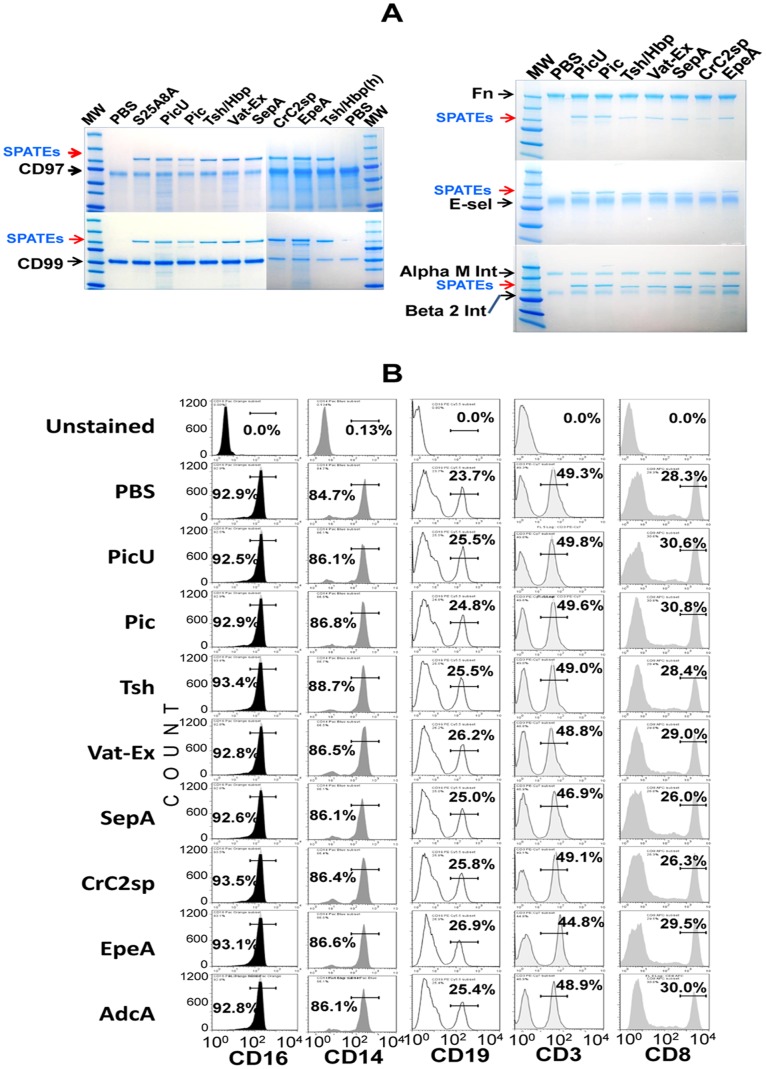
Class-2 SPATEs do not degrade non mucin-type, O-linked glycoproteins. **A**, In order to assess the proteolytic activity of class-2 SPATEs on other heavily glycosylated proteins, we treated CD97, CD99, E-selectin, αβ-integrin and fibronectin with 2 uM class-2 SPATEs by overnight incubation at 37°C. Samples were analyzed by SDS-PAGE and Blue Commassie staining. **B**, Proteolytic activity of SPATEs was also evaluated on leukocyte markers routinely used in flow cytometry assays. 1×10^6^ total leukocytes were treated with 2 uM of SPATEs for 1 h at 37°C and analyzed by multi-color flow cytometry. The data show cleavage profiles against CD16(used in NK gating), CD14(monocytes), CD3(T lymphocytes), CD8(Cytotoxic lymphocytes) and CD19(B lymphocytes), and are representative of more than 5 independent experiments.

In our previous study we found that the Pic protease activity was dependent on mucin-type O-glycosylation [11]. In order to determine whether other glycoprotein types are also susceptible to SPATE proteolytic activity, we incubated CD97 and CD99 [26], [27]; two non-mucin type glycoproteins, and three heavily N-linked glycosylated proteins such as the fibronectin, E-selectin and the alpha (M)beta (2) integrin with SPATEs overnight at 37°C and analyzed them by SDS-PAGE for proteolytic cleavage. Under these conditions, none of the these molecules were susceptible to SPATEs (Figure 5A). Furthermore, SPATE proteins had no effect on leukocyte molecules such as CD16, CD14, CD19, CD3 and CD8 as assessed by flow cytometry (Figure 5B).

### Class-2 SPATEs target glycoproteins on all hematopoietic lineage

**Figure 6:**
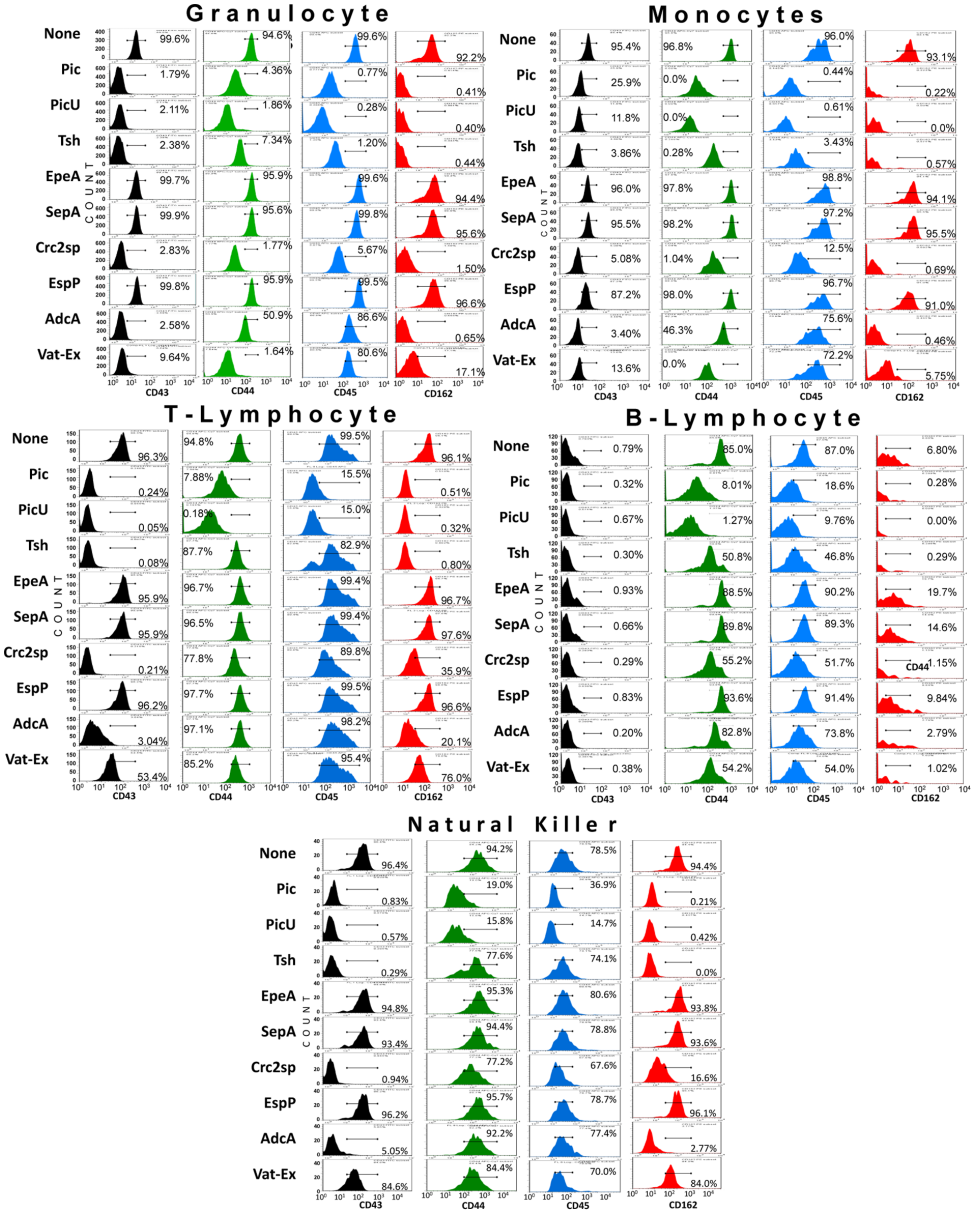
Class-2 SPATEs degrades the extracellular domain of mucin-type O-glycoproteins on human leukocytes subpopulation. 1×10^6^ total leukocytes isolated from human blood were incubated with 2 µM of purified SPATEs at 37°C for 1 h. Multi-color flow cytometry was used to analyze cleavage of glycopoteins in different leukocyte subpopulations. Cell types were identified through “forward and side scatter” gates plus cell type-specific lineage markers. Monocytes, T, and B lymphocytes were concurrently stained with conjugated antibodies directed against CD14, CD3 and CD19, respectively. Granulocytes were selected by gating. NK cells were first negatively selected for binding of anti-CD3 and then positively selected for binding of anti–CD16 and anti–CD56. The data show cleavage profiles of CD43, CD44, CD45 and CD162, and are representative of 3 independent experiments.

**Figure 7:**
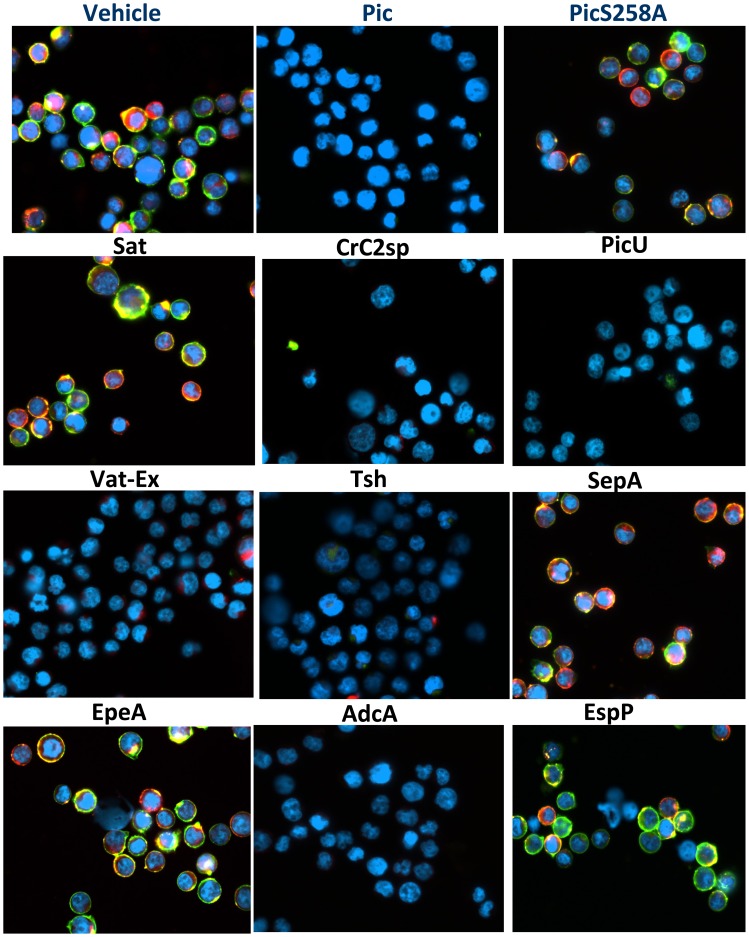
Cleavage of CD43 and CD162 glycoproteins in Jurkat T cells by class-2 SPATEs. To visualize degradation of mucin-type glycoproteins by SPATEs, Jurkat T cells were treated with 2 µM of each SPATE for 1 h at 37°C. T cells were stained using fluorescent monoclonal antibodies against human CD43 (green) and CD162 (red) simultaneously, and analyzed by fluorescence microscopy. CD43 shows up in green, CD162 in red and co-localization of both, CD43 and CD162 is in yellow. DAPI was used to visualize cell nucleus (blue). Micrographs were taken at 40× magnification.

To evaluate the proteolytic activity of SPATEs on native leukocyte glycoproteins, we isolated white cells from healthy donors and incubated them with equimolar concentration of SPATEs for 1 h at 37°C, followed by assessment of cleavage by flow cytometry. Specific cell types and glycoproteins were identified through scatter gates, cell type-specific lineage markers, and dye-conjugated antibodies specific to SPATE-susceptible glycoproteins such as CD43, CD44, CD45 and CD162. As expected, most SPATEs except EpeA, SepA or the non-class-2 SPATE EspP were able to cleave glycoproteins on the surface of granulocytes, monocytes (CD+14) and T(CD3+), B(CD19+) and natural killer(CD3−, CD16+, CD56+) lymphocytes (Figure 6). Overall, we observed efficient cleavage of O-linked glycoproteins in all cell populations, however the cleavage efficiency was more pronounced on granulocytes and monocytes than the lymphoid populations. Also, the cleavage pattern of CD162 and CD43 were in agreement with the extensive cleavage of these proteins seen by SDS-PAGE with recombinant proteins. Interestingly, the AdcA SPATE protein which lacks of “Domain-2”; characteristic in other class-2 SPATE members, cleaved glycoproteins in all cell populations suggesting that domain-2 is not involved in the glycoprotease activity (Figure 6). Pic displayed the most efficient protease activity and AdcA the lesser; the overall performance was: Pic>PicU> Tsh/Hbp> CrC2sp> Vat-Ex> AdcA (Figure 4 and 6). Loss of CD43 and CD162 extracellular domains following T cell treatment with SPATEs was also confirmed by fluorescence microscopy (Figure 7).

### Reduced glycoprotease activity on deglycosylated substrates

**Figure 8:**
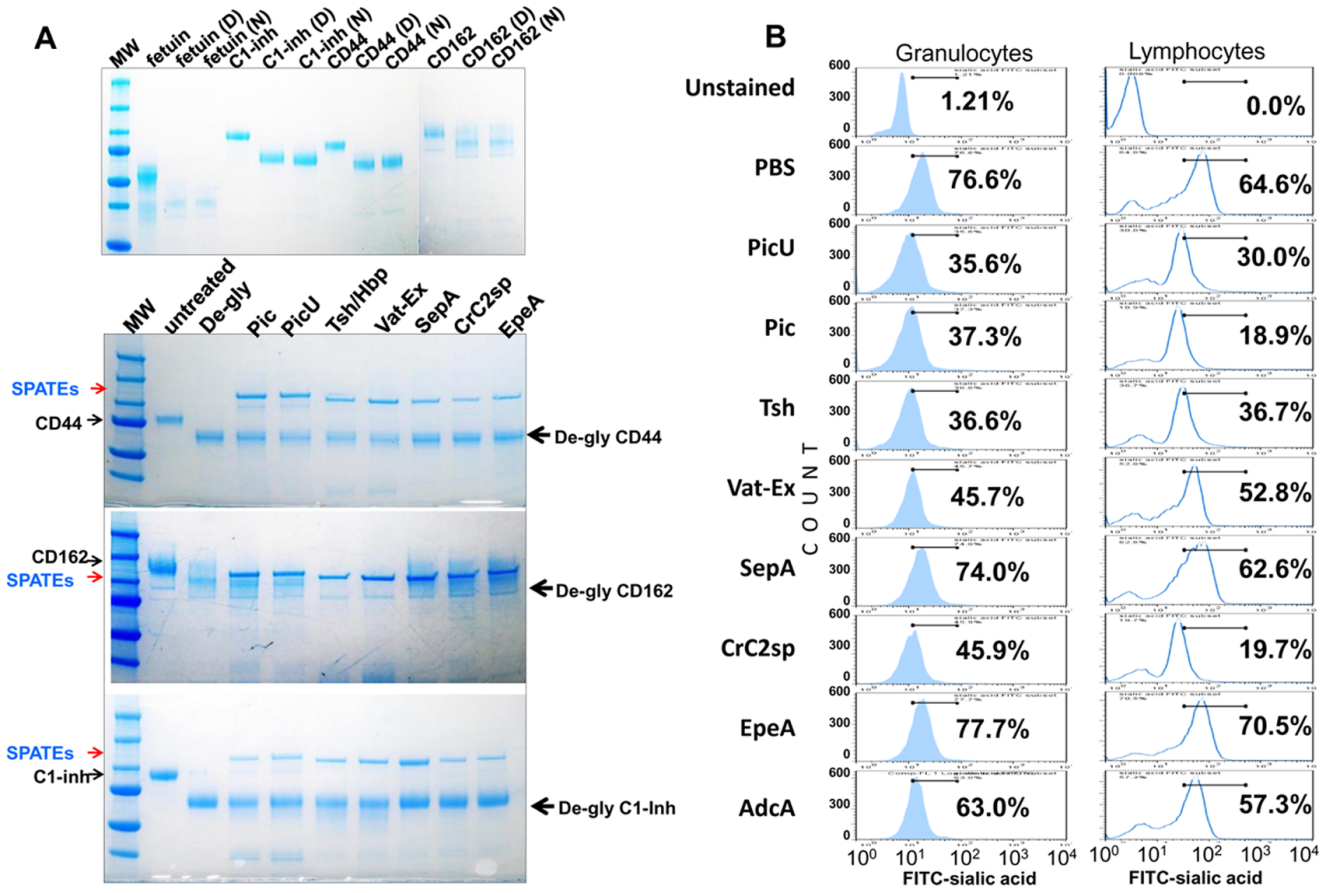
Reduced proteolytic activity of SPATE on deglycosylated proteins. **A**, Upper panel; purified CD44, CD162 and C1-Inh glycoproteins were deglycosylated with deglycosylation enzyme cocktail by overnight at 37°C under denaturing (D) and non-denaturing (N) conditions. Extent of deglycosylation was assessed by comparison with untreated glycoproteins and by mobility shifts on SDS-PAGE gels. Lower panel; 1 µg of non-denaturing deglycosylated proteins were treated with 2 uM of each class-2 SPATE by 2 h at 37°C and analyzed by SDS-PAGE and blue Commassie staining. **B**, The overall sialic acid carbohydrate depletion on human leukocytes following treatment with 2 µM class-2 SPATEs during 1 h at 37°C was assessed by flow cytometry. Data show the percentage of sialic acid-bearing granulocytes and lymphocyte populations, and are representative of three independent experiments.

We and other groups have previously shown that Pic activity on glycoproteins relies on the saccharide nature of substrates. Pic displays reduced proteolytic activity on deglycosylated substrates, and reduced binding ability to mucin in presence of free monosaccharides [11], [28]. In order to determine if other class-2 members share this property, we treated CD44, CD162 and C1-inh with a deglycosylation enzyme cocktail followed by incubation with class-2 SPATEs for two hours at 37°C (Figure 8A). We observed reduced cleavage of deglycosylated proteins by all class-2 SPATEs when compared with intact glycoproteins treated for only one hour (Figure 4 and 8A). Although Tsh/Hbp and Vat-Ex showed reduced protease activity on deglycosylated CD44 and C1-inh, they efficiently cleaved deglycosylated CD162 (Figure 8A), which seemed to be partially deglycosylated, as judged by the presence of bands with higher molecular weight similar to the glycosylated protein (Figure 8A). Crc2sp showed lesser proteolytic activity on deglycosylated proteins than other class-2 SPATE. Pic and PicU showed similar activity on deglycosylated proteins, with some degree of degradation of higher molecular weight species of deglycosylated CD162. However, all deglycosylated proteins were degraded after overnight incubation with class-2 SPATEs except SepA and EpeA (data not shown).

### Overall reduction of leukocyte surface carbohydrates by SPATEs

Removal of O-linked glycoproteins by SPATEs must also be reflected in loss of cell surface carbohydrates. To test this, we assessed the amount of sialic acid on the surface of leukocytes following SPATE treatment by flow cytometry. We observed more than 50% depletion of the sialic acid-bearing granulocytes and T cell populations after one hour treatment with class-2 SPATEs (Figure 8B).

### Single amino acid mutations in domain-1 of Pic protease abolishes glycoprotease activity

**Figure 9:**
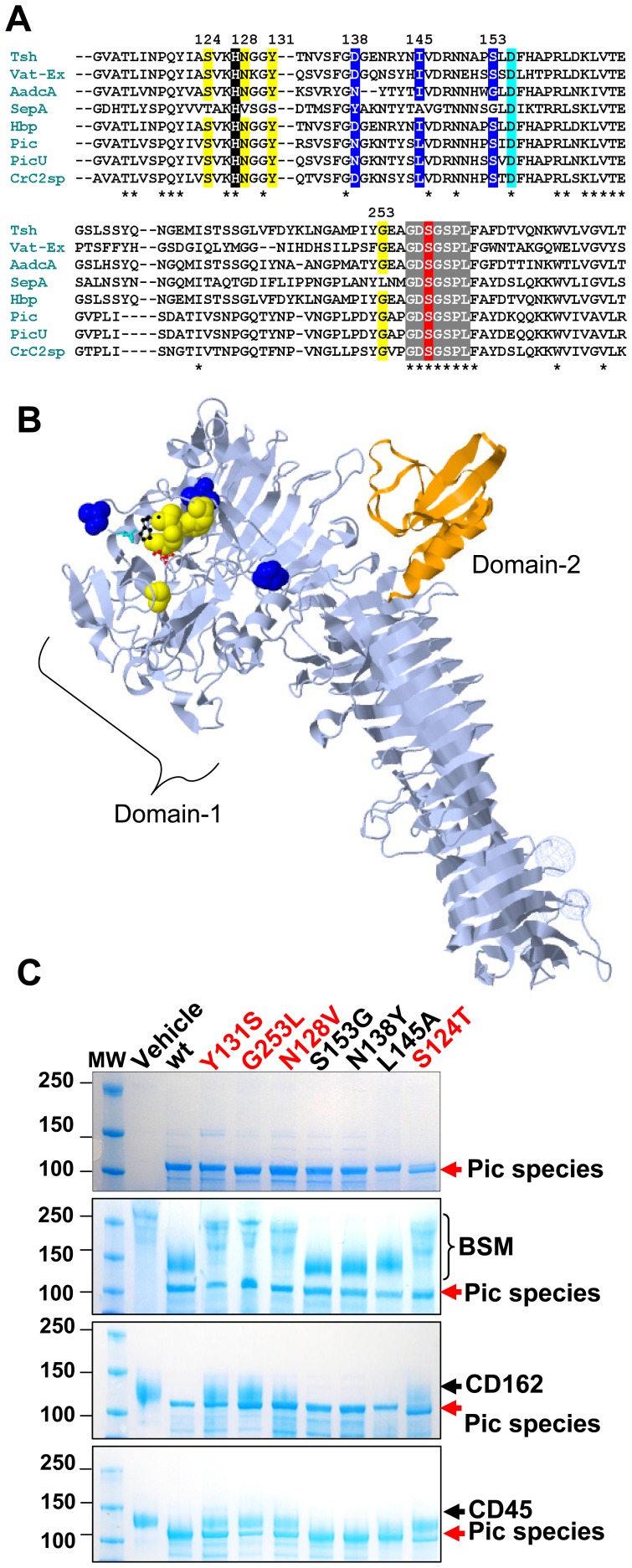
Conserved residues in the region spanning the catalytic triad of class-2 SPATEs are important for glycoprotease activity. A, The aminoacid sequence of the region spanning the catalytic triad in domain-1 of class-2 SPATEs, were aligned with Clustal-omega. The residues comprising the catalytic triad are shaded in black (His), cyan (D) and red (S). The conserved catalytic motif of SPATEs are shaded in grey. Aminoacids shaded in yellow (conserved in most class-2) and blue (non conserved in all SPATEs) were mutated to those occurring in SepA by site directed mutagenesis. B, Positions of mutated residues (spheres) with respect to the catalytic triad (little spheres and sticks) in Pic, are depicted in the stereo ribbon structure of Hbp (PDB, WXR) [15]. In orange, the domain-2 characteristic in most class-2 SPATEs, but not in AdcA is shown. C, Secretion of Pic derivatives harboring single site mutations and their activity on glycoproteins. Concentrated supernatant proteins from HB101 expressing Pic species were analyzed by SDS-PAGE and Commassie staining (top gel). 3 ug of glycoproteins (BSM, CD162, and CD45) were incubated with 10 µL of each supernatant for 2 h at 37°C, and analyzed as above (bottom gels). BSM, bovine submaxillary mucin.

Alignment of the amino acid sequence of domain-1 (harboring the catalytic serine protease triad) of SepA, with all other class-2 SPATEs with glycoprotease activity using Clustal-Omega [29], revealed very conserved residues among class-2 members which were not shared with SepA (Figure 9A). To determine the relevance of those residues in glycoprotease activity, we mutated seven randomly selected residues in Pic, spanning the catalytic triad, to the residues naturally occurring in SepA by site directed mutagenesis (Figure 9A–C). Surprisingly, we found that even though secretion of the constructs harboring site mutations was not affected, mutation of conserved residues greatly reduced the glycoprotease activity of Pic on glycoproteins (Figure 9C).

## Discussion and Conclusions

Pathogenic *E. coli* and *Shigella* are a significant cause of morbidity and mortality worldwide. They have been classified into pathotypes based in the clinical, pathological and epidemiological features of the disease they cause. Despite pathotypes harbor many diverse virulence factors, they all have in common the SPATEs, whose contributions have remained until recently obscure. We observed that most members of the class-2 SPATE family have the ability to agglutinate leukocytes, here evidenced in Jurkat T cells. The agglutination phenotype resembled that observed in a number of lectins produced by plants and microorganisms [30]. In addition, Tsh/Hbp and Pic have been previously shown to agglutinate red cells. Interestingly, we observed that the agglutinating activity appears rapidly upon contact with leukocytes, and increased over the time of exposure (Data not shown), but was dependent on the serine protease activity given that denatured Pic protease or the protease-deficient PicS258A failed to agglutinate leukocytes. Likewise, Sat, a class-1 SPATE, did not exhibit this phenotype.

We observed direct binding of most class-2 SPATEs to all leukocyte populations including granulocytes, monocytes, T, B and natural killer lymphocytes. Interestingly, binding efficiency was higher in granulocytes and monocytes than lymphocytes. We have previously shown that Pic protease activity against glycoproteins is reliant on the saccharide modification of the substrates since treatment of these substrates with neuraminidase greatly reduced or inhibited Pic protease activity [11]. The binding patterns could be explained by the fact that in leukocytes of the innate immune system such as the granulocytes, the glycosyltransferases involved in formation of the O-glycans are constitutively expressed, while in T cells, they are expressed only after appropriate activation. Moreover, it is known that B-cells express very low number of glycoproteins (such as CD43 and CD162) in the resting state [31], [32], in agreement with our results (Figure 6). Surprisingly, we observed high binding capacity of the protease deficient PicS258A, but not by the heat-denatured Pic protease or SepA, suggesting that the agglutination phenotype is dependent on protease and not on binding activity.

We recently showed that Pic and Tsh/Hbp but not SepA were able to cleave O-linked glycoproteins such as CD43, CD44, CD45, CD93, CD162 and CX3CL1. In fact, in this study we show that most class-2 SPATEs except SepA and EpeA cleaved virtually all mucin-type O-linked glycoproteins, but not other non mucin-type, heavily glycosylated proteins such as E-selectin, αβ-integrin or fibronectin. In addition to previously reported substrates, we found that components of the complement system such as C1-INH and CD55, adhesion proteins CD34 and CD164, and members of the recently discovered Tim family [T cell, immunoglobulin and mucin domain] glycoproteins; Tim1, Tim3 and Tim4 were susceptible to SPATE digestion. Although most SPATEs cleaved mucin type-glycoproteins they show different proteolytic strengths, having Pic, its allelic variant PicU and its closest homologue CrC2sp produced by *C. rodentium*, as the most proteolytically efficient SPATEs, followed by Tsh/Hbp and its closer homologues Vat-Ex and AdcA. This latter, differs from other class-2 SPATEs in that AdcA does not possess domain-2 (Figure 9B); characteristic in all other members. We found the processed form of the AdcA passenger protein mostly associated to the bacterium, but concentrated supernatants from HB101 expressing AdcA was able to cleave all glycoproteins tested including native glycoproteins in human primary leukocytes (Figure 6).

We also observed that glycosylation of substrates greatly potentiate SPATE proteolytic activity, judged by the fact that reduced SPATE activity was observed on deglycosylated proteins. We noticed variable protease activity throughout all SPATEs on deglycosylated proteins, which may be the consequence of aminoacid variation in the binding-catalytic pocket site (domain-1, Figure 9B) of SPATEs and/or incomplete removal of complex *O*-linked glycans on deglycosylated substrates.

When the crystal structure of the first class-2 protease was solved it was hypothesized that domain-2 contributed to substrate recognition, but the lack of known biological substrates made difficult to address this proposition. The fact that AdcA, a domain-2-less SPATE cleaves glycoproteins, suggests that domain-2 does not play a role in substrate recognition.

Our purified SPATEs cleaved O-glycoproteins in both recombinant and native forms and in all leukocyte types. The extent of proteolytic activity was comparable to the binding activity observed in early experiments, with higher glycoprotein degradation in cells involved in the innate immune response, but also significantly in lymphocytes, including natural killer cells. It is predictable that cleavage of glycoproteins in lymphocytes may increase upon cell activation, where the expression level of glycoproteins and the enzymes involved in glycosylation processes are steadily enhanced. In fact, we previously observed that activated T cells treated with Pic undergo apoptosis, but not resting T cells [11].

SepA and EpeA, which are distantly located in the phylogenetic tree from other class-2 SPATEs (Figure 1), showed no glycoprotease activity on leukocytes, however, just recently a closer homologue of SepA; EatA, was shown to degrade other O-glycoproteins, such as those lining the intestinal mucosa, including Muc2 [33]. It is tempting to hypothesize that SepA deviated from the other class-2 SPATEs in such a way that the glycoprotease activity was narrowed to only those glycoproteins present in the intestinal mucosa. In fact, when we compared the amino acid sequence of domain-1 (where the H-D-S catalytic triad is located) among class-2 SPATEs, we found that many residues spanning the catalytic triad were conserved in all proteolytic active SPATEs, but not in SepA. We found that changing single residues in Pic for those naturally occurring in SepA, significantly reduced glycoprotease activity. Since the introduction of site mutations in Pic did not affect its secretion, and the fact that SepA is still proteolytically active in other substrates [13], this finding suggests that lectin activity is encoded in the region spanning the catalytic triad in domain-1.

In conclusion, we have here described a family of serine proteases with lectin-like properties, whose main role may lie in their ability to modulate the immune response at various levels. SPATEs cleave chemokines, complement proteins, adhesion proteins and co-stimulatory molecules, all involved in malignancy and inflammatory processes. We recognize that the broad specificity and functionality of class-2 SPATEs, in both innate and adaptive arms of the immune system suggests new therapeutic strategies against a large number of inflammatory disorders (patent # PCT/US2010/061758).

## Supporting Information

Figure S1 **Leukocyte subpopulations identified in whole blood by FACS analysis.** The characteristic forward (FSC) and side (SSC) scatter profile was used to distinguish lymphocytes, monocytes and granulocytes. Within the lymphocyte population, B lymphocytes, T lymphocytes, and NK cells were identified using membrane markers CD19, CD3 and CD16/CD56, respectively. NK cells were first negatively selected for binding of anti–CD3 and then positively selected for binding anti–CD16 and anti–CD56. Monocytes were CD14+ and neutrophils were selected only by gating on granulocytes
(TIF)

## References

[1] Page MJ, Di Cera E (2008) Serine peptidases: classification, structure and function. Cell Mol Life Sci 65: 1220–1236. 18259688 10.1007/s00018-008-7565-9 PMC11131664

[2] Ruiz-Perez F, Nataro JP (2013) Bacterial serine proteases secreted by the autotransporter pathway: classification, specificity, and role in virulence. Cell Mol Life Sci. 10.1007/s00018-013-1355-8 PMC3871983 23689588

[3] Yen YT, Kostakioti M, Henderson IR, Stathopoulos C (2008) Common themes and variations in serine protease autotransporters. Trends Microbiol 16: 370–379. 18595714 10.1016/j.tim.2008.05.003

[4] Leyton DL, Rossiter AE, Henderson IR (2012) From self sufficiency to dependence: mechanisms and factors important for autotransporter biogenesis. Nat Rev Microbiol 10: 213–225. 22337167 10.1038/nrmicro2733

[5] Henderson IR, Navarro-Garcia F, Nataro JP (1998) The great escape: structure and function of the autotransporter proteins. Trends Microbiol 6: 370–378. 9778731 10.1016/s0966-842x(98)01318-3

[6] Gomis SM, Riddell C, Potter AA, Allan BJ (2001) Phenotypic and genotypic characterization of virulence factors of Escherichia coli isolated from broiler chickens with simultaneous occurrence of cellulitis and other colibacillosis lesions. Can J Vet Res 65: 1–6. 11227188 PMC1189634

[7] Otto BR, van Dooren SJ, Nuijens JH, Luirink J, Oudega B (1998) Characterization of a hemoglobin protease secreted by the pathogenic Escherichia coli strain EB1. J Exp Med 188: 1091–1103. 9743528 10.1084/jem.188.6.1091 PMC2212542

[8] Henderson IR, Czeczulin J, Eslava C, Noriega F, Nataro JP (1999) Characterization of pic, a secreted protease of Shigella flexneri and enteroaggregative Escherichia coli. Infect Immun 67: 5587–5596. 10531204 10.1128/iai.67.11.5587-5596.1999 PMC96930

[9] Navarro-Garcia F, Gutierrez-Jimenez J, Garcia-Tovar C, Castro LA, Salazar-Gonzalez H, et al (2010) Pic, an autotransporter protein secreted by different pathogens in the Enterobacteriaceae family, is a potent mucus secretagogue. Infect Immun 78: 4101–4109. 20696826 10.1128/IAI.00523-10 PMC2950354

[10] Harrington SM, Sheikh J, Henderson IR, Ruiz-Perez F, Cohen PS, et al (2009) The Pic protease of enteroaggregative Escherichia coli promotes intestinal colonization and growth in the presence of mucin. Infect Immun 77: 2465–2473. 19349428 10.1128/IAI.01494-08 PMC2687332

[11] Ruiz-Perez F, Wahid R, Faherty CS, Kolappaswamy K, Rodriguez L, et al (2011) Serine protease autotransporters from Shigella flexneri and pathogenic Escherichia coli target a broad range of leukocyte glycoproteins. Proc Natl Acad Sci U S A 108: 12881–12886. 21768350 10.1073/pnas.1101006108 PMC3150873

[12] Bestebroer J, Poppelier MJ, Ulfman LH, Lenting PJ, Denis CV, et al (2007) Staphylococcal superantigen-like 5 binds PSGL-1 and inhibits P-selectin-mediated neutrophil rolling. Blood 109: 2936–2943. 17132726 10.1182/blood-2006-06-015461

[13] Dutta PR, Cappello R, Navarro-Garcia F, Nataro JP (2002) Functional comparison of serine protease autotransporters of enterobacteriaceae. Infect Immun 70: 7105–7113. 12438392 10.1128/IAI.70.12.7105-7113.2002 PMC133081

[14] Hart E, Yang J, Tauschek M, Kelly M, Wakefield MJ, et al (2008) RegA, an AraC-like protein, is a global transcriptional regulator that controls virulence gene expression in Citrobacter rodentium. Infect Immun 76: 5247–5256. 18765720 10.1128/IAI.00770-08 PMC2573378

[15] Otto BR, Sijbrandi R, Luirink J, Oudega B, Heddle JG, et al (2005) Crystal structure of hemoglobin protease, a heme binding autotransporter protein from pathogenic Escherichia coli. J Biol Chem 280: 17339–17345. 15728184 10.1074/jbc.M412885200

[16] Simmons DL, Satterthwaite AB, Tenen DG, Seed B (1992) Molecular cloning of a cDNA encoding CD34, a sialomucin of human hematopoietic stem cells. J Immunol 148: 267–271. 1370171

[17] Watt SM, Chan JY (2000) CD164–a novel sialomucin on CD34+ cells. Leuk Lymphoma 37: 1–25. 10721766 10.3109/10428190009057625

[18] Kane LP (2010) T cell Ig and mucin domain proteins and immunity. J Immunol 184: 2743–2749. 20200285 10.4049/jimmunol.0902937 PMC3069641

[19] Cai S, Davis AE 3rd (2003) Complement regulatory protein C1 inhibitor binds to selectins and interferes with endothelial-leukocyte adhesion. J Immunol 171: 4786–4791. 14568956 10.4049/jimmunol.171.9.4786

[20] Coyne KE, Hall SE, Thompson S, Arce MA, Kinoshita T, et al (1992) Mapping of epitopes, glycosylation sites, and complement regulatory domains in human decay accelerating factor. J Immunol 149: 2906–2913. 1383332

[21] Clark MC, Baum LG (2012) T cells modulate glycans on CD43 and CD45 during development and activation, signal regulation, and survival. Ann N Y Acad Sci 1253: 58–67. 22288421 10.1111/j.1749-6632.2011.06304.x PMC4190024

[22] Carlow DA, Gossens K, Naus S, Veerman KM, Seo W, et al (2009) PSGL-1 function in immunity and steady state homeostasis. Immunol Rev 230: 75–96. 19594630 10.1111/j.1600-065X.2009.00797.x

[23] Bazan JF, Bacon KB, Hardiman G, Wang W, Soo K, et al (1997) A new class of membrane-bound chemokine with a CX3C motif. Nature 385: 640–644. 9024663 10.1038/385640a0

[24] Park M, Tenner AJ (2003) Cell surface expression of C1qRP/CD93 is stabilized by O-glycosylation. J Cell Physiol 196: 512–522. 12891708 10.1002/jcp.10332

[25] Bennett KL, Modrell B, Greenfield B, Bartolazzi A, Stamenkovic I, et al (1995) Regulation of CD44 binding to hyaluronan by glycosylation of variably spliced exons. J Cell Biol 131: 1623–1633. 8522617 10.1083/jcb.131.6.1623 PMC2120678

[26] Wang J, Shiratori I, Satoh T, Lanier LL, Arase H (2008) An essential role of sialylated O-linked sugar chains in the recognition of mouse CD99 by paired Ig-like type 2 receptor (PILR). J Immunol 180: 1686–1693. 18209065 10.4049/jimmunol.180.3.1686 PMC2577149

[27] Hsiao CC, Cheng KF, Chen HY, Chou YH, Stacey M, et al (2009) Site-specific N-glycosylation regulates the GPS auto-proteolysis of CD97. FEBS Lett 583: 3285–3290. 19737555 10.1016/j.febslet.2009.09.001

[28] Gutierrez-Jimenez J, Arciniega I, Navarro-Garcia F (2008) The serine protease motif of Pic mediates a dose-dependent mucolytic activity after binding to sugar constituents of the mucin substrate. Microb Pathog 45: 115–123. 18538533 10.1016/j.micpath.2008.04.006

[29] Sievers F, Wilm A, Dineen D, Gibson TJ, Karplus K, et al (2011) Fast, scalable generation of high-quality protein multiple sequence alignments using Clustal Omega. Mol Syst Biol 7: 539. 21988835 10.1038/msb.2011.75 PMC3261699

[30] Souza MA, Carvalho FC, Ruas LP, Ricci-Azevedo R, Roque-Barreira MC (2013) The immunomodulatory effect of plant lectins: a review with emphasis on ArtinM properties. Glycoconj J 30: 641–657. 23299509 10.1007/s10719-012-9464-4 PMC3769584

[31] Laszik Z, Jansen PJ, Cummings RD, Tedder TF, McEver RP, et al (1996) P-selectin glycoprotein ligand-1 is broadly expressed in cells of myeloid, lymphoid, and dendritic lineage and in some nonhematopoietic cells. Blood 88: 3010–3021. 8874199

[32] Wiken M, Bjorck P, Axelsson B, Perlmann P (1988) Induction of CD43 expression during activation and terminal differentiation of human B cells. Scand J Immunol 28: 457–464. 3264083 10.1111/j.1365-3083.1988.tb01476.x

[33] Kumar P, Luo Q, Vickers TJ, Sheikh A, Lewis WG, et al. (2013) EatA, an Immununogenic Protective Antigen of Enterotoxigenic Escherichia coli Degrades Intestinal Mucin. Infect Immun. 10.1128/IAI.01078-13 PMC3911389 24478066

