# Supplementary figures and images for: Broad Spectrum Activity of a Lectin-Like Bacterial Serine Protease Family on Human Leukocytes
Source: PLoS One. 2014 Sep 24;9(9):e107920. doi: 10.1371/journal.pone.0107920 (PMC4176022; doi:10.1371/journal.pone.0107920)

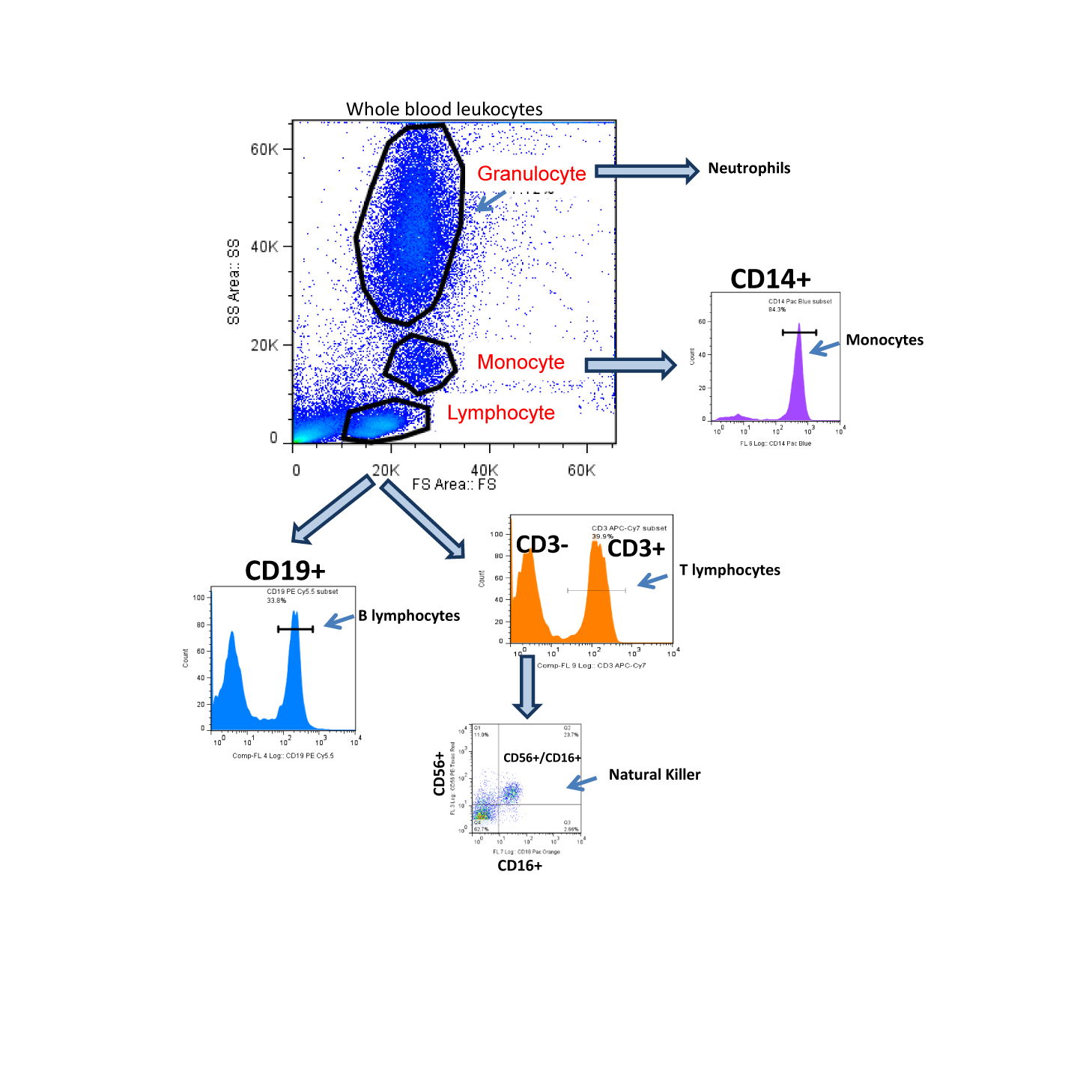

Supplement: Figure S1 — Leukocyte subpopulations identified in whole blood by FACS analysis. The characteristic forward (FSC) and side (SSC) scatter profile was used to distinguish lymphocytes, monocytes and granulocytes. Within the lymphocyte population, B lymphocytes, T lymphocytes, and NK cells were identified using membrane markers CD19, CD3 and CD16/CD56, respectively. NK cells were first negatively selected for binding of anti–CD3 and then positively selected for binding anti–CD16 and anti–CD56. Monocytes were CD14+ and neutrophils were selected only by gating on granulocytes (TIF) [file pone.0107920.s001.tif]
